# Fibrolamellar Variant of Hepatocellular Carcinoma in a Young Female

**DOI:** 10.7759/cureus.16486

**Published:** 2021-07-19

**Authors:** Kevin Walsh, Niranjan Ojha, Rahul Seth

**Affiliations:** 1 Internal Medicine, State University of New York Upstate Medical University, Syracuse, USA; 2 Hematology and Oncology, State University of New York Upstate Medical University, Syracuse, USA

**Keywords:** fibrolamellar, hepatocellular carcinoma (hcc), hepatomegaly, unintentional weight loss, hepatic tumors

## Abstract

Fibrolamellar variant of hepatocellular carcinoma (FLHCC) is a rare malignancy. Our patient presented to the hospital with increasing weight loss, decreased appetite, and night sweats. Imaging revealed a large liver mass and multiple pulmonary nodules concerning for metastasis. The patient eventually underwent interventional radiology (IR)-guided biopsy of the liver which revealed pathology consistent with FLHCC. The patient was discharged from the hospital and was scheduled for a follow up at an outpatient cancer center close to her family.

## Introduction

Cancer is a leading cause of death worldwide, accounting for nearly 10 million deaths in 2020 [1]. The most common causes of cancer death in 2020 were lung, colon, liver, stomach, and breast cancer [1]. Cancer is a disease in which some of the body's cells grow uncontrollably and can even involve other parts of the body [2]. Human cells grow and multiply through a process called cell division, and when these cells become old, they eventually die [2]. This process can become altered in which damaged cells multiply and continue to divide when they should not and eventually become tumors [2]. Tumors can either be benign or malignant. Benign tumors are slow-growing tumors that do not spread throughout the body [3]. Malignant tumors are tumors which invade the surrounding tissue and can spread throughout the body via the blood or lymphatic system [3]. The particular cancer we discuss in our case is fibrolamellar variant of hepatocellular carcinoma (FLHCC). FLHCC is a rare tumor comprising less than 1% of all primary liver tumors in the United States [4]. Epidemiologically, this cancer affects younger individuals under 40 years of age and equally affects males and females (cancer). Currently, there are no environmental or lifestyle risk factors for acquiring fibrolamellar carcinoma. Typically, cirrhosis and hepatitis are not risk factors for FLHCC, unlike hepatocellular carcinoma [5]. The gold standard for FLHCC diagnosis is a liver biopsy, and better outcomes are usually seen in patients who are candidates for resection [5].

## Case presentation

A 29-year-old female with a past medical history significant for low-grade squamous intraepithelial lesion s/p colposcopy and laser therapy in 2017 presented to the hospital with worsening upper abdominal pain, nausea, night sweats, weight loss, and decreased appetite for approximately three weeks duration. Approximately three weeks ago, the patient noticed she started to become very nauseated after meals. She thought her nausea was due to poor diet as she was eating an excessive amount of ice cream. The patient was unsure of how much weight she lost over the past several weeks, but her family stated that her weight loss was noticeable. The patient was recently deployed to Afghanistan and returned home in January 2020 after completing a tour.

**Figure 1:**
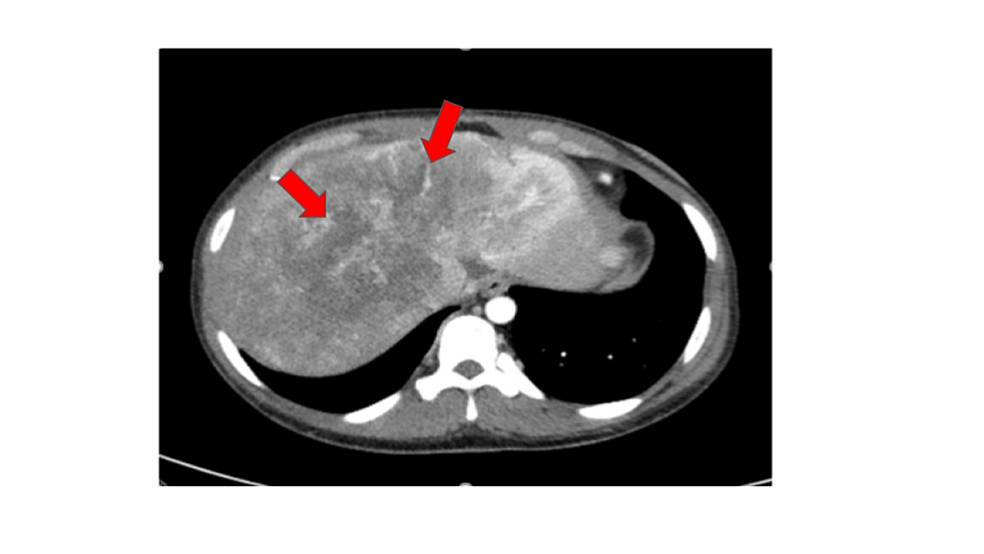
CT of the abdomen/pelvis revealing massive hepatomegaly and very large bilobed central hepatic mass measuring 19.3 cm x 10 cm x 9.4 cm. Arrows pointing toward bilobed central hepatic mass.

On physical exam, the patient was afebrile and hypotensive with blood pressure 104/73. The abdominal exam was remarkable for tenderness to palpation in the right upper quadrant. Laboratory values were remarkable for microcytic anemia, anion gap of 18, alkaline phosphatase 139, aspartate aminotransferase 76, alanine aminotransferase 74, total protein 7.3, total bilirubin 0.3, CA125 47 U/mL (normal <38), CA 19-9 15.6 U/mL (normal <35). Ultrasound abdomen was completed, which revealed diffuse gallbladder wall thickening up to 7 mm, trace scattered free fluid in the right upper quadrant, and a large heterogeneous mass in the right lobe of the liver, measuring 10.7 x 9.5 x 12.7 cm. Ultrasound also revealed a large mass seen towards the midline, measuring 10.4 cm maximally. CT abdomen pelvis revealed massive hepatomegaly, bilateral pulmonary nodules, very large bilobed central hepatic mass measuring 19.3 cm x 10 cm x 9.4 cm, and one enlarged portocaval lymph node below the hepatic porta (Figure 1). CT thorax revealed multiple scattered bilateral pulmonary metastases measuring up to 15 mm. CT head and MRI brain were unremarkable for any acute pathology.

Due to presentation and imaging findings, hepatobiliary surgery, gastroenterology, and obstetrics and gynecology (OBGYN) were initially consulted. Gynecology reasoned that the mildly elevated CA125 could be from the known liver mass and did not indicate ovarian malignancy. Further, an epithelial, ovarian neoplasm, or germ cell tumor was unlikely given the lack of adnexal mass on imaging. Gynecology also reasoned that patient’s history of low-grade abnormality on Pap smear three years ago was improbable to have progressed to cervical cancer as on exam cervix was grossly normal. HB surgery recommended that the patient needed interventional radiology (IR)-guided liver biopsy to evaluate further and identify liver mass.

The patient underwent an IR-guided liver biopsy, and pathology revealed well-differentiated, large, polygonal tumor cells with eosinophilic hyaline cytoplasmic bodies and abundant fibrous stroma arranged in thin parallel lamellae around tumor cells consistent with hepatocellular carcinoma with features most suggestive of FLHCC.

Due to pathology results, hematology/oncology was consulted and recommended the patient to undergo IR-guided biopsy of lung nodules found on imaging as this would guide treatment. The patient was discharged with a plan for an outpatient lung biopsy. 

## Discussion

Fibrolamellar hepatocellular carcinoma is a rare liver cancer that affects adolescents and young adults with no history of primary liver disease [4]. The tumor was first defined in 1956 and is a variant of hepatocellular carcinoma [5]. Patients tend to have normal alpha fetoprotein levels as opposed to elevated levels found in hepatocellular carcinoma [5]. As mentioned previously, FLHCC tends to affect individuals under 40 years of age, and there are no environmental or lifestyle risk factors for acquiring FLHCC [5]. Little information is available on the molecular pathogenesis leading to the development of FLHCC, and currently, research is still in progress to determine the most efficacious chemotherapy. Patients who are appropriate candidates for surgical resection tend to have better outcomes [5].

When symptoms are present, they are generally nonspecific and usually include abdominal pain, malaise, and fatigue [6]. In our case, the patient presented with these similar nonspecific symptoms, as mentioned previously, in addition to night sweats and weight loss. Patients with FLHCC usually blame their symptoms on other common conditions. Our patient blamed her symptoms on poor diet as she stated on presentation that she was eating a large amount of ice cream daily in recent weeks.

Microscopic evaluation of the tumor reveals well-differentiated polygonal hepatic cells with eosinophilic and granular cytoplasm surrounded by thick, fibrous stroma arranged in bands [5]. Appropriate candidates for surgical resection include candidates who do not have metastatic spread, major vessel involvement, or extensive nodal spread. Patients who undergo liver resection have a median five-year survival of 40%-70%. Poorer outcomes are noted for patients with extrahepatic disease who cannot undergo resection with a five-year survival of 2%-15% [1]. Systemic therapy is an appropriate option for patients with unresectable tumors, but there is no consistently effective form of systemic therapy, and no trials are supporting the benefit of any regimen over another.

In regards to tumor pathogenesis, Honeyman et al. (2014) investigated the molecular basis of FLHCC and identified a chimeric transcript that was expressed in FLHCC but not in normal liver. This chimeric transcript resulted due to a deletion on chromosome 19 and was found to code for a protein containing DNAJB1 (DnaJ/HSP40 homolog, subfamily B, member 1) and PRKACA (catalytic domain of protein kinase A). Honeyman et al. (2014) performed western blot analyses and immunoprecipitation, which confirmed that the chimeric protein was expressed in tumor tissue in 15/15 patients with FLHCC, indicating a potential role of DNAJB1 and PRKACA in tumor pathogenesis. The role of the fusion protein is still being investigated at the time of this publication [7]. More recently, DNAJB1 PRKACA fusion protein has also been discovered in other malignancies of pancreaticobiliary origin, making the recognition of this protein less sensitive for FLHCC.

Stipa et al. (2006) performed a prospective study published in the American Cancer Society in 2006 on patients with FLHCC referred to a tertiary care center over 18 years from 1986-2003. The median age of all patients was 27 years, and 28 patients underwent complete gross resection while 13 patients had an unresectable disease. The median tumor size was 9 cm in those who underwent resection, and 36% of these patients had vascular invasion while 50% had lymph node metastasis. The study found that at 34 months, five-year survival was 76% for resected patients. Median survival for those who did not undergo resection was 12 months. Six of the patients who did not undergo resection were deemed unresectable due to extensive liver involvement or peritoneal metastases. The other seven were deemed unresectable based on preoperative imaging. In the study, 17 patients underwent a second operation after a median interval of 37 months due to recurrence. The median survival after the second operation was 26 months [5].

Chakrabarti et al. (2019) performed a retrospective study by chart reviewing 42 patients with FLHCC treated between 1990-2017 at the Mayo Clinic [8]. All ten patients with stage I disease and 21 of 32 patients with stage II-IVB underwent resection; 6/10 patients in the stage I group developed recurrence with a median time to recurrence of 30.5 months. The five-year overall survival (OS) was 86% in the stage I group. Patients with stage II to IVB disease who underwent resection upfront had a median OS of 32.5 months and a five-year OS of 44%. In the upfront surgery group, 71% of patients experienced recurrence. The median OS of patients with the unresectable disease (n=11) was ten months. This study revealed that surgical resection was associated with prolonged overall survival; however, there was significant reoccurrence.

## Conclusions

FLHCC remains a rare tumor worldwide, and research is ongoing to determine potential risk factors, patient presentation, and effective treatment strategies. From the studies published regarding patient outcomes, it is evident that patients have prolonged survival if their disease is amenable to resection; however, reoccurrence is common. Our patient presented with a typical presentation of the disease and is unlikely to be a resection candidate, given the likely pulmonary involvement. Unfortunately, the patient was discharged before the biopsy of the pulmonary nodules.
